# Anesthetic management of a patient with chronic inflammatory demyelinating polyneuropathy by combination of total intravenous and regional anesthesia

**DOI:** 10.1186/s40981-019-0241-2

**Published:** 2019-03-09

**Authors:** Daiki Takekawa, Kishiko Nakai, Hirotaka Kinoshita, Junichi Saito, Masato Kitayama, Tetsuya Kushikata, Kazuyoshi Hirota

**Affiliations:** 1 Department of Anesthesiology, Hirosaki University Graduate School of Medicine, 5 Zaifu-cho, Hirosaki, 036-8562 Japan; 2 Division of Surgical Center, Hirosaki University Hospital, 53 Hon-cho, Hirosaki, 036-8563 Japan

**Keywords:** Chronic inflammatory demyelinating polyneuropathy, Total intravenous anesthesia, Regional anesthesia

## Abstract

Chronic inflammatory demyelinating polyneuropathy (CIPD) is a rare acquired immune-mediated progressive and relapsing disorder that causes peripheral neuropathy lasting more than 2 months. We report the successful anesthetic management of a 66-year-old man with CIPD undergoing the laparoscopic Hartmann procedure. We induced and maintained total intravenous anesthesia (TIVA) using propofol, remifentanil, and ketamine without muscle relaxants. We performed ultrasound-guided transversus abdominis plane and rectus sheath blocks with 60 ml of 0.25% levobupivacaine for achieving good surgical conditions. For postoperative analgesia, we intravenously administered fentanyl (200 μg) and acetaminophen (1000 mg). The patient was uneventfully extubated in the operating room after confirming adequate spontaneous breathing. The postoperative course was uneventful without any respiratory complications such as respiratory depression, aspiration pneumonia, or progression of CIPD symptoms.

## Background

Chronic inflammatory demyelinating polyneuropathy (CIPD) is a rare acquired immune-mediated progressive and relapsing disorder that causes peripheral neuropathy lasting more than 2 months [1]. The classic presentation of CIDP includes slow progression of both proximal and distal muscle weakness often accompanied by sensory deficits [2]. Only few reports on the anesthetic management of patients with CIPD exist due to the rarity of the disease, and a standard safe anesthetic management has not been established. The anesthetic management of patients with CIPD is challenging due to the prolonged effects of muscle relaxants in them and the resulting increased risk of postoperative respiratory complications. Thus, a combination of general and regional anesthesia may be one of the best options because regional anesthesia can induce abdominal relaxation and reduce opioid doses. Here, we report on the successful anesthetic management of a patient with CIPD undergoing the laparoscopic Hartmann procedure using a combination of total intravenous anesthesia (TIVA) and ultrasound-guided abdominal wall blocks.

## Case presentation

We obtained a written informed consent from the patient for the publication of this case report.

The patient was a 66-year-old man (165 cm, 62 kg) with a history of CIPD diagnosed several years ago. He was taking prescribed oral cyclosporine (75 mg per day). Because of muscle weakness in the upper and lower limbs and right and left hand grip strengths being 10 kg and 6 kg, respectively, he walked with the help of a walker and managed to have his meals using a spoon. He required some assistance with his activities of daily living.

The patient was found to have anemia in a periodic blood test, and he was diagnosed with rectal cancer following a detailed examination. Therefore, he was scheduled to undergo the laparoscopic Hartmann procedure. His respiratory function tests showed that he had restrictive ventilatory impairment (VC, 2470 ml; %VC, 66.9%; FEV_1.0,_ 1910 ml; %FEV_1.0,_ 77.0%). He did not have any other abnormal medical history or laboratory results.

On the morning of the surgery, the patient was administered roxatidine (75 mg) as anesthetic premedication. We induced anesthesia using propofol (80 mg), ketamine (30 mg), and remifentanil (0.3 μg/kg/min), and maintained it using propofol (4–5 mg/kg/h) and remifentanil (0.15–0.2 μg/kg/min) without the use of muscle relaxants. Tracheal intubation was uneventful with a 1% lidocaine 4 ml tracheal sprinkle. After the induction, we performed the ultrasound-guided transversus abdominis plane and rectus sheath blocks using 60 ml of 0.25% levobupivacaine. Additionally, we administered fentanyl (200 μg) and acetaminophen (1000 mg) intravenously for postoperative analgesia. The abdominal wall blocks helped in maintaining good surgical conditions without the use of muscle relaxants. The operation lasted 4 h and 25 min and the total blood loss was a little. The patient emerged from the anesthesia and was uneventfully extubated in the operating room after confirming adequate spontaneous breathing. Next, the patient was transferred to the intensive care unit and was administered a continuous intravenous infusion of fentanyl (12.5 μg/h) for postoperative analgesia. On the following day, we discontinued the continuous intravenous infusion of fentanyl and moved the patient to the general ward. The postoperative course was uneventful without any respiratory complications such as respiratory depression, aspiration pneumonia, and progression of CIPD symptoms.

## Discussion

No specific guidelines for the anesthetic management of patients with CIPD exist due to the rarity of the disease. However, caution must be exercised due to the prolonged effect of muscle relaxants and the increased risk for postoperative respiratory complications and disease exacerbation in patients with CIPD. Thus, we chose general and regional anesthesia without the use of muscle relaxants.

Patients with some types of neuromuscular disorders are markedly sensitive to muscle relaxants [3]. In fact, Hara and colleagues reported prolonged effects of vecuronium in a patient with CIPD undergoing partial gastrectomy [4]. However, Maruyama and colleagues reported the safe use of rocuronium with sugammadex for reversal while monitoring muscle relaxation in three patients with CIPD [5], and Tezcan and colleagues used sugammadex successfully to reverse rocuronium in a patient with Guillain-Barre syndrome (GBS), which is a disease similar to CIPD [6]. Hence, muscle relaxant reversals with sugammadex may be safely used in patients with CIPD. On the contrary, confirmed cases of allergic reactions to clinical doses of sugammadex have been reported, and the sensitivity of patients with CIPD to muscle relaxants requires further research. Therefore, we preferred to avoid the use of muscle relaxants and sugammadex in the present case.

For inducing abdominal relaxation without muscle relaxants, regional anesthesia is required. However, the safety of regional anesthesia for CIPD patients has not been established. Wiertlewski and colleagues reported the case of a woman with GBS in whom the neurologic status worsened after labor under epidural analgesia [7]. Conversely, Bhaskar et al. successfully managed a patient with CIPD undergoing cystolithotripsy by spinal anesthesia [8]. Additionally, Richter T et al. stated that spinal anesthesia is acceptable for cesarean delivery in patients with CIDP, although partial neural blocks in the feet persisted for 1 day [9]. To the best of our knowledge, no reports have described peripheral nerve blocks for patients with CIPD. However, we chose peripheral nerve blocks instead of an epidural block and spinal anesthesia after considering the surgical approach and the fact that our patient was compromised by immunosuppressants. Our patient did not experience postoperative progression of CIPD symptoms including abdominal sensory deficits, and we were able to reduce the opioid doses. Thereby, the uneventful postoperative course proceeded without any respiratory complications. However, further data are required to establish the safety of peripheral nerve blocks for patients with CIPD.

## Conclusion

In summary, we successfully managed the anesthesia of a patient with CIPD undergoing laparoscopic surgery using a combination of TIVA with ultrasound-guided abdominal wall blocks without the use of muscle relaxants.

## Abbreviations

CIPD: Chronic inflammatory demyelinating polyneuropathy
GBS: Guillain-Barre syndrome
TIVA: Total intravenous anesthesia

## References

[1] Bright RJ, Wilkinson J, Coventry BJ (2014). Therapeutic options for chronic inflammatory demyelinating polyradiculoneuropathy: a systematic review. BMC Neurol.

[2] Reynolds J, Sachs G, Stavros K (2016). Chronic inflammatory demyelinating polyradiculoneuropathy (CIDP): clinical features, diagnosis, and current treatment strategies. R I Med J (2013).

[3] Schmitt HJ, Muenster T (2009). Anesthesia in patients with neuromuscular disorders. Minerva Anestesiol.

[4] Hara K, Minami K, Takamoto K, Shiraishi M, Sata T (2000). The prolonged effect of a muscle relaxant in a patient with chronic inflammatory demyelinating polyneuropathy. Anesth Analg.

[5] Maruyama N, Wakimoto M, Inamori N, Nishimura S, Mori T (2015). Anesthetic management of three patients with chronic inflammatory demyelinating polyradiculoneuropathy. Masui.

[6] Tezcan B, Bölükbaşi D, Kazanci D, Turan S, Suer Kaya G, Özgök A (2017). The use of sugammadex in a patient with Guillain-Barre syndrome: a case report. A A Case Rep.

[7] Wiertlewski S, Magot A, Drapier S, Malinovsky JM, Péréon Y (2004). Worsening of neurologic symptoms after epidural anesthesia for labor in a Guillain-Barré patient. Anesth Analg.

[8] Bhaskar SB, Srinivasalu D (2016). Intrathecal dexmedetomidine for anaesthetic management of a patient with chronic inflammatory demyelinating polyneuropathy. J Clin Diagn Res.

[9] Richter T, Langer KA, Koch T (2012). Spinal anesthesia for cesarean section in a patient with chronic inflammatory demyelinating polyradiculoneuropathy. J Anesth.

